# Supplementary material for: Requirements engineering issues causing software development outsourcing failure
Source: PLoS One. 2020 Apr 9;15(4):e0229785. doi: 10.1371/journal.pone.0229785 (PMC7144980; doi:10.1371/journal.pone.0229785)
Supplement: S1 Appendix — (DOCX) [file pone.0229785.s003.docx]

**S3 Appendix A. Consolidated list of RE process issues in case of SDO**

| **IDs** | **(1): Literature-based Communication Issues of RE Process for SDO** |
| --- | --- |
| Iss1 | Occasional and controlled correspondence amongst the shareholders [40]. |
| Iss2 | Deficiency of casual correspondence amongst the shareholders [33, 91–93]. |
| Iss3 | To explain and resolve the confusions regarding requirements, person to person correspondence is essential [94]. |
| Iss4 | Deficiency of person to person correspondence [93, 95]. |
| Iss5 | Deficiency of synchronized correspondence [96–97]. |
| Iss6 | Even via the videoconferences, it is difficult to enable extensive and fruitful dialogs specifically in case of numerous shareholders [98]. |
| Iss7 | Deferred replies [93, 99–100]. |
| Iss8 | Planning the co-located gatherings amongst shareholders is impractical mostly as shareholders are detached [101–102]. |
| Iss9 | There is improper correspondence between customer and vendor [69]. |
| Iss10 | Organizing person to person get-togethers heightens the cost [21, 97, 101]. |
| Iss11 | Shareholders do not utilize synchronized Internet communication technologies to convey information regarding requirements, instead rely on traditional approached alike planned meetings, electronic mails and documentation [92]. |
| Iss12 | The gatherings that are held for making decisions regarding requirements are fruitless [28,33]. |
| Iss13 | Asynchronous correspondence leads to deferment in proliferation and resolution of issues [102]. |
| Iss14 | If there are synchronous meetings amid the locations which have substantial differences regarding time then participants belonging to some locations are bothered as there are huge differences between the meeting times and their local working times [40, 102–103]. |
| Iss15 | Shareholders are not able to express in the correspondence language [33]. |
| Iss16 | Electronic correspondence alike email permits clandestine correspondence that generates complications for settling clashes regarding requirements [33]. |
| Iss17 | Shareholders don't convey to one another adequately, instead seek to apply force and utilize influence on one another [102]. |
| Iss18 | To illuminate and resolve the issues, any coworker may correspond with any shareholder that may cause tedious debates and additional controlling endeavors [104]. |
| Iss19 | Correspondence gaps or postponements amid RE because of individuality conflicts [105]. |
| Iss20 | Online correspondence to elucidate requirements prompts spiny requirements because resulting requirements are uncertain, alter again and again or are unfinished [106]. |
| Iss21 | To arrange interviews, acquiring the assent of far off shareholders [107]. |
| **IDs** | **Additional Communication Issues of RE Process for SDO Reported by SDO Practitioners** |
| Iss22 | Typically, there is non-recording of the promises that are done amid videoconferencing or discussions on the telephone, consequently such pledges cannot be alluded when needed [Proposed]. |
| **IDs** | **(2):Literature-based** [**Knowledge Management & Awareness**](http://www.um.es/giisw/GSD/wiki/index.php/Knowledge_management_and_awareness) **Issues of RE Process for SDO** |
| Iss23 | Obstacles in flow of requirements information towards organizations or from organization [108]. |
| Iss24 | Ineptitude of keeping track of the shareholders, and related data, who are influenced because of the introduction of novel requirements [109]. |
| Iss25 | Shareholders are incompetent to look for pertinent information, strategies are coordinated improperly to incorporate the information, and information exchange is deferred or blocked [110]. |
| Iss26 | Unfamiliarity of the shareholders from existing/recent data regarding requirements [111]. |
| Iss27 | Requirements data attained by various far off origins is not imparted to every shareholder [28, 40, 103]. |
| Iss28 | Physically dispersed shareholders are unable to receive the rewards of communal mechanisms and procedures that are available for collocated workspace, consequently, need for consciousness regarding the requirements is increased [112]. |
| Iss29 | Reviving of the previously conversed and apparently resolved issues [38, 113]. |
| Iss30 | Inappropriate allocation of duties, with respect to administrative organization, may hamper the circulation of information [114]. |
| Iss31 | Proliferation of the data regarding requirements modifications is inadequate [92]. |
| Iss32 | Professionals inadvertently neglect to apprise pertinent shareholders regarding the modifications in the requirements [92]. |
| Iss33 | The professionals’ clusters engaged in the similar or related requirements are unaware about the shareholders who are influenced by requirements modifications or who stimulate the requirements modifications [92]. |
| Iss34 | Inadequate management of the modifications in requirements [69, 115]. |
| Iss35 | The diversified bunches engaged in similar or linked requirements are uninformed regarding the specialists of the far-off teams [92]. |
| Iss36 | Traditional sources for correspondence alike documents are unable to reveal the alterations in requirements as fast as needed [112, 116]. |
| Iss37 | Functioning on the outdated requirements [111, 117]. |
| Iss38 | Hitches in accessibility of the steady data because of the dissemination of sources [118]. |
| Iss39 | Scarcity of the mindfulness regarding deployment environment may cause ambiguity in requirements [94]. |
| Iss40 | Unfamiliarity to the background and significance of requirements can cause project postponements and quality tradeoffs [119]. |
| Iss41 | Requirements illuminations are passed on later than expected time which can cause project postponements [111]. |
| Iss42 | Incapability to share information or finest practices [28, 120]. |
| Iss43 | Requirements engineers are ignorant of the impacts of novel system deployment upon customer’s organization [121]. |
| **IDs** | **Additional** [**Knowledge Management & Awareness**](http://www.um.es/giisw/GSD/wiki/index.php/Knowledge_management_and_awareness) **Issues of RE Process for SDO Reported by SDO Practitioners** |
| Iss44 | The professional bunches engaged in similar or related requirements do not know which requirement is being addressed by whom [Proposed]. |
| Iss45 | Unfamiliarity with or not consulting all the origins of requirements [Proposed]. |
| Iss46 | Inappropriate tracking of the requirements [Proposed]. |
| **IDs** | **(3): Literature-based Cultural Diversities Issues of RE Process for SDO** |
| Iss47 | Detachment leads to cultural variances amongst the different working departments belonging to an organization which produces difficulty in achieving the shared awareness about the requirements [33, 102]. |
| Iss48 | Generating trust amongst the different shareholders is demanding [33, 107, 122–125]. |
| Iss49 | Upholding trust amongst the different shareholders is demanding [123, 125]. |
| Iss50 | Scarcity of trust amongst the different shareholders [17, 93, 107, 122–123, 126]. |
| Iss51 | Evasion of the obligations from the different shareholders [94]. |
| Iss52 | Forfeiture of attachment amongst the shareholders on account of physical dispersal [127]. |
| Iss53 | Complications in attaining consent on requirements [30, 40, 94, 128]. |
| Iss54 | Shareholders originate from miscellaneous social backgrounds and own dissimilar moral standards regarding hierarchies, addressing risks, tracking timetables and promptness that can intensify disagreements [94]. |
| Iss55 | Various cultures follow dissimilar values concerning exactness of work done and capability of inventiveness [118]. |
| Iss56 | Professionals from differing social foundations have ambiguous and implicit implications and clarifications of the data about the requirements [39, 129]. |
| Iss57 | Professionals from different social foundations derive mixt implications from messages [130]. |
| Iss58 | A few experts, due to their social foundations, cannot do disagreement with the customers, hence, ‘pleasing’ requirements and main requirements are assigned same preferences [118]. |
| Iss59 | Requirements of the client are not completely comprehended and conveyed due to divergent cultural foundations and languages [131]. |
| Iss60 | Contributors of the far-off gatherings, regarding requirements engineering, are not skilled in sole communication language [97, 132]. |
| Iss61 | Shareholders are at various capability level of the correspondence language, consequently, shareholders at advanced level influence and dominate the correspondence about requirements [100]. |
| Iss62 | Identical words are utilized to pass on the dissimilar implications in various associations that generates confusions for requirements description and approval [33]. |
| Iss63 | The persons, not capable in correspondence language, are hesitant in making inquiries for requirements elucidations [100]. |
| Iss64 | Bashfulness of the shareholders, for instance evading from doing telephone calls to unacquainted individuals, causes deferred correspondence [101]. |
| Iss65 | The requirements cognizance is diminished in case of describing the requirements in the non- indigenous language [94]. |
| **IDs** | **Additional Cultural Diversities Issues of RE Process for SDO Reported by SDO Practitioners** |
| Iss66 | Noninvolvement or elimination of shareholders during RE related events [Proposed]. |
| Iss67 | A portion of the stakeholders do not take part in the RE associated discussions in view of their non-familiarity with the correspondence language [Proposed]. |
| Iss68 | Challenges to set the practical assumptions regarding reply time [Proposed]. |
| **IDs** | **(4): Literature-based Management and Coordination Issues of RE Process for SDO** |
| Iss69 | Complications in grasping evidences, motives and actions needed for mutual Requirements Understanding (RU) amongst the scattered shareholders [29, 33, 102]. |
| Iss70 | Disparities in the regional-times of the stakeholders create hinderance in synchronizing RE associated events [133-134]. |
| Iss71 | Obstruction for contribution of shareholders in RE related events due to time contrasts [40]. |
| Iss72 | Postponement in elucidations regarding requirements and finalizing decisions [94]. |
| Iss73 | Tendency of not-mentioning RE-related issues due to remoteness [103]. |
| Iss74 | Even the skillful experts can end up anxious and dormant on account of being far off [105]. |
| Iss75 | Improperly defined or vague obligations [118, 135]. |
| Iss76 | Absenteeism of pivotal and reliable administration for RE process that origins improper coordination [105]. |
| Iss77 | Absenteeism of a steady, talented and focal analyst role [105]. |
| Iss78 | Underrating the time needed for performing requirements appraisal [105]. |
| Iss79 | Discriminating distribution of working load to different groups [136]. |
| Iss80 | No evaluation of the impact of shareholders' dissemination on various RE related tasks [136]. |
| Iss81 | Contradictory benefits of various shareholders [30, 33]. |
| Iss82 | Requirements obtained from the distributed shareholders belonging to different hierarchical units, are needed to be bundled [135]. |
| Iss83 | Requirements are obtained from the huge number of shareholders [118]. |
| Iss84 | Genuine requirements are needed to be altered to interface with different software systems [135]. |
| Iss85 | Requirements are modified by analyst by overlooking the recommended procedure [105]. |
| Iss86 | Given the time-based dispersal, harmonized coordination is needed to generate the trust [134]. |
| Iss87 | Distant RE groups work with confined timetable to fulfill deadlines [5, 137]. |
| **IDs** | **Additional** [**Management and Coordination**](http://www.um.es/giisw/GSD/wiki/index.php/Management_and_project_coordination) **Issues of RE Process for SDO Reported by SDO Practitioners** |
| Iss88 | Group fellow(s) expect that other group fellow(s) have to accomplish similar obligations [Proposed]. |
| Iss89 | Failure in performing RE associated assignment(s) as everyone believes this is obligation of another person [Proposed]. |
| Iss90 | Impractical resource division to accomplish RE [Proposed]. |
| **IDs** | **(5): Literature-based Processes and Tools Issues of RE Process for SDO** |
| Iss91 | Absence of obviously delineated RE process [94, 136]. |
| Iss92 | The shareholders utilize divergent procedures for examining and recording requirements [92]. |
| Iss93 | Shareholders utilize diverse procedures to conduct alterations in requirements [92]. |
| Iss94 | The standard RE procedures are not followed [105, 118]. |
| Iss95 | Utilization of various RE procedures introduces various formats and techniques at distant sites of customer [26, 136]. |
| Iss96 | Utilizing inappropriate RE procedures [118]. |
| Iss97 | Some group fellows don't participate in RE consultations because they are unfamiliar with the apparatuses and techniques being utilized [138]. |
| Iss98 | The instruments can't be merged with different instruments [118]. |
| Iss99 | RE associated rework or information loss amid exchanges among various tools [26]. |
| Iss100 | Necessity for the instruments that give perpetual access to data associated with requirements [117]. |
| Iss101 | Instruments don't pass on data, about requirements change, to the pertinent shareholders at the suitable time [139]. |
| Iss102 | Necessity for the instruments that enable the discernibility of requirements crosswise the fringes of instruments [117]. |
| Iss103 | Necessity for the instruments that assist requirements dialogs amongst the distant shareholders [140]. |
| Iss104 | Incapability of the tools for evolving the requirements documents by enabling coordination amongst the distant shareholders [139]. |
| Iss105 | Choosing the unsuitable RE instrument(s) [26, 118]. |
| Iss106 | Scarcity of coaching for utilizing groupware instruments [127]. |
| **IDs** | **Additional Processes and Tools Issues of RE Process for SDO Reported by SDO Practitioners** |
| Iss107 | Utilization of inadequate technique for eliciting requirements [Proposed]. |
| Iss108 | Assumptions regarding instruments and Technologies are not fulfilled [proposed]. |
| Iss109 | The instruments have security and scalability problems [Proposed]. |
| **IDs** | **(6): Literature-based issues originated from Relationship among stakeholders** |
| Iss110 | Absence of steady relationship amongst the shareholders [93, 141]. |
| Iss111 | Not passing on data, to identify or settle requirements related issues, to dispersed locations for a longer time span [92]. |
| Iss112 | Rarity of casual interactions leads to fewer chances of establishing relations [100]. |
| Iss113 | Utilization of various standards, by client and vendor, for documenting the requirements [26]. |
| Iss114 | Creation of client or/and service provider teams on temporary base [26]. |
| Iss115 | Disparate preferences of customer and vendor to collect and confirm requirements [26]. |
| Iss116 | Less involvement of customer side during requirements engineering process [26, 33]. |
| Iss117 | Team(s) from vendor side have misapprehensions regarding working practices of the client side [26]. |
| Iss118 | Customer and vendor pursue contradictory approaches for requirements engineering [26]. |
| Iss119 | Unsuccessfulness of vendor to meet due dates and satisfy the obligations regarding requirements [26]. |
| Iss120 | Problems of deciding about requirements related deliverables [26]. |
| Iss121 | Disagreement on choice of RE instruments [26]. |
| Iss122 | Clients feel that executing requirements associated work from distant requirements is impassible [21]. |
| Iss123 | Customer and service provider depend on verbal contract [105]. |
| **IDs** | **(7): Literature-based Requirements centric issues of RE process for SDO** |
| Iss124 | Confirming requirements in case of all shareholders relying on the requirements collected or data acquired only from the accessible shareholders [129]. |
| Iss125 | Requirements’ descriptions are misunderstood [69, 142]. |
| Iss126 | Inaccurate or wrong requirements [143]. |
| Iss127 | Not creating the requirements founded on suitable business cases [144]. |
| Iss128 | Gold-plated or additional requirements [144]. |
| Iss129 | Uncompleted requirements [109, 137, 143]. |
| Iss130 | No standards for documenting the requirements [145]. |
| Iss131 | Inclusion of the requirements that are not within the scope [135]. |
| Iss132 | Requirements are described/specified ambiguously [5, 21, 69, 109, 118, 146]. |
| Iss133 | Not giving data or giving deliberately vague data about requirements [33, 102]. |
| Iss134 | Non availability of the criterion to prioritize the requirements [118]. |
| Iss135 | Requirements are altered again and again [5, 69, 109, 146]. |
| Iss136 | Discrepancies in the requirements related documents [109]. |
| Iss137 | Enlarging the requirements that causes scope slinking [5]. |
| Iss138 | Requirements are elicited via fragmentation, means various individuals finalize the requirements belonging to various system’s fragments, that causes client displeasure [147]. |
| Iss139 | Analysts are devoid of the tactics that are needed to address the requirements description issues in case of outsourced projects [105]. |
| Iss140 | Just chosen shareholders are counseled to elicit the requirements that roots for prejudiced elicitation [148]. |
| Iss141 | Actual end users and individuals who collaborate with the analysts are not same [121]. |
| Iss142 | Analysts are influenced to conceal certain data associated to requirements that grounds for compromises to elicit and describe the requirements [121]. |
| **IDs** | **Additional Requirements centric Issues of RE process for SDO Reported by SDO Practitioners** |
| Issu143 | Clients are uncertain regarding the software requirements [Proposed]. |
| Iss144 | Analysts presume, in view of their expertise, that they are aware of the clients’ requirements [Proposed]. |
| Iss145 | Clients are intrigued by the services provided by various systems and desire that their system should provide similar facilities, however, actually they are not needed [Proposed]. |
| Issu146 | Customers emphasis on including more requirements whereas cost and schedule have been settled [Proposed]. |
| Iss147 | Absence of real clients currently [Proposed]. |
| Iss148 | Employing a technique to elicit requirements but its appropriateness is not investigated [Proposed]. |
| Iss149 | General approach to address the problem is incorrect [Proposed]. |
| Iss150 | Applying presumptions to confirm or conclude requirements [Proposed]. |
