# Supplementary material for: Requirements engineering issues causing software development outsourcing failure
Source: PLoS One. 2020 Apr 9;15(4):e0229785. doi: 10.1371/journal.pone.0229785 (PMC7144980; doi:10.1371/journal.pone.0229785)
Supplement: S2 Appendix — (DOCX) [file pone.0229785.s004.docx]

**S4 Appendix B. Average frequency & standard deviation, for each issue, calculated after 2^nd^ & 3^rd^ Delphi rounds**

| **Sr. #** | **Issue IDs** | **Round 2** | | **Round 3** | |
| --- | --- | --- | --- | --- | --- |
|  |  | **Average** | **St. Dev.** | **Average** | **St. Dev.** |
|  | Iss1 | 4.077670 | .7629833 | 4.116505 | .7316510 |
|  | Iss2 | 4.194175 | .7417289 | 4.203883 | .7324311 |
|  | Iss3 | 1.592233 | .6632790 | 1.563107 | .5886159 |
|  | Iss4 | 1.689320 | .6864105 | 1.669903 | .6004630 |
|  | Iss5 | 4.106796 | .7658472 | 4.126214 | .7628586 |
|  | Iss6 | 1.485437 | .6695635 | 1.485437 | .6240927 |
|  | Iss7 | 4.203883 | .7456965 | 4.213592 | .7363195 |
|  | Iss8 | 1.941748 | .8612035 | 1.912621 | .8057002 |
|  | Iss9 | 1.679612 | .7031246 | 1.660194 | .6191930 |
|  | Iss10 | 1.504854 | .6697056 | 1.495146 | .6242452 |
|  | Iss11 | 1.543689 | .6970062 | 1.533981 | .6540303 |
|  | Iss12 | 4.058252 | .7647278 | 4.097087 | .6790213 |
|  | Iss13 | 1.679612 | .6295593 | 1.660194 | .5866722 |
|  | Iss14 | 1.553398 | .6527191 | 1.543689 | .6067706 |
|  | Iss15 | 1.650485 | .6960496 | 1.640777 | .6080243 |
|  | Iss16 | 1.737864 | .8039262 | 1.728155 | .7435233 |
|  | Iss17 | 1.679612 | .6449440 | 1.660194 | .6031518 |
|  | Iss18 | 1.504854 | .6083373 | 1.504854 | .5920020 |
|  | Iss19 | 1.543689 | .6827956 | 1.543689 | .6382682 |
|  | Iss20 | 1.825243 | 2.0264292 | 1.796117 | 2.0017601 |
|  | Iss21 | 1.495146 | .6397577 | 1.495146 | .6242452 |
|  | Iss22 | 4.174757 | .6920725 | 4.194175 | .6868264 |
|  | Iss23 | 3.844660 | .7765848 | 3.854369 | .7721598 |
|  | Iss24 | 1.563107 | .6366256 | 1.553398 | .5895854 |
|  | Iss25 | 1.543689 | .7109328 | 1.504854 | .6242452 |
|  | Iss26 | 4.165049 | .7933188 | 4.165049 | .7933188 |
|  | Iss27 | 1.631068 | .6102120 | 1.631068 | .5939283 |
|  | Iss28 | 1.786408 | .7363195 | 1.766990 | .7029892 |
|  | Iss29 | 4.077670 | .7368364 | 4.087379 | .7290444 |
|  | Iss30 | 1.553398 | .6820982 | 1.504854 | .6242452 |
|  | Iss31 | 1.766990 | .7695668 | 1.766990 | .7567200 |
|  | Iss32 | 1.679612 | .6746617 | 1.640777 | .5916804 |
|  | Iss33 | 1.514563 | .6396089 | 1.504854 | .6242452 |
|  | Iss34 | 4.135922 | .7547048 | 4.165049 | .7553351 |
|  | Iss35 | 1.543689 | .6534479 | 1.533981 | .5910365 |
|  | Iss36 | 1.660194 | .6649989 | 1.631068 | .6102120 |
|  | Iss37 | 4.048544 | .8674805 | 4.077670 | .8247319 |
|  | Iss38 | 1.504854 | .6397577 | 1.504854 | .6083373 |
|  | Iss39 | 1.660194 | .7078467 | 1.631068 | .6415406 |
|  | Iss40 | 1.524272 | .6393112 | 1.514563 | .6240927 |
|  | Iss41 | 1.961165 | .7786657 | 1.961165 | .7530634 |
|  | Iss42 | 2.009709 | .8459262 | 2.000000 | .8284169 |
|  | Iss43 | 4.067961 | .7829323 | 4.077670 | .7757264 |
|  | Iss44 | 1.475728 | .5915195 | 1.485437 | .6240927 |
|  | Iss45 | 4.097087 | .7860869 | 4.106796 | .7785434 |
|  | Iss46 | 1.631068 | .6714090 | 1.601942 | .6158018 |
|  | Iss47 | 1.553398 | .6059858 | 1.543689 | .5903920 |
|  | Iss48 | 1.504854 | .6549029 | 1.485437 | .6240927 |
|  | Iss49 | 1.524272 | .6393112 | 1.514563 | .5918412 |
|  | Iss50 | 3.970874 | .8795756 | 4.009709 | .8342561 |
|  | Iss51 | 3.970874 | .8219574 | 4.000000 | .7669650 |
|  | Iss52 | 1.631068 | .5939283 | 1.640777 | .6080243 |
|  | Iss53 | 3.990291 | .9234966 | 4.009709 | .9020146 |
|  | Iss54 | 1.524272 | .6237876 | 1.514563 | .5918412 |
|  | Iss55 | 1.640777 | .6546121 | 1.621359 | .6122364 |
|  | Iss56 | 1.495146 | .5920020 | 1.504854 | .5920020 |
|  | Iss57 | 1.514563 | .6081808 | 1.514563 | .6240927 |
|  | Iss58 | 1.592233 | .6778989 | 1.543689 | .6067706 |
|  | Iss59 | 1.495146 | .6242452 | 1.495146 | .6242452 |
|  | Iss60 | 1.533981 | .6388644 | 1.514563 | .5918412 |
|  | Iss61 | 1.834951 | .7289139 | 1.815534 | .6965964 |
|  | Iss62 | 1.514563 | .6695635 | 1.495146 | .6242452 |
|  | Iss63 | 1.543689 | .6382682 | 1.524272 | .5915195 |
|  | Iss64 | 1.689320 | .6421338 | 1.669903 | .6004630 |
|  | Iss65 | 1.533981 | .7251171 | 1.504854 | .6397577 |
|  | Iss66 | 3.961165 | .8623080 | 3.970874 | .8569933 |
|  | Iss67 | 1.669903 | .6165742 | 1.660194 | .6031518 |
|  | Iss68 | 3.980583 | .8964044 | 4.019417 | .8964044 |
|  | Iss69 | 4.058252 | .7899521 | 4.077670 | .7882634 |
|  | Iss70 | 1.543689 | .6227186 | 1.524272 | .5915195 |
|  | Iss71 | 1.504854 | .6242452 | 1.495146 | .6242452 |
|  | Iss72 | 4.145631 | .7721598 | 4.165049 | .7682050 |
|  | Iss73 | 1.572816 | .6355782 | 1.553398 | .5895854 |
|  | Iss74 | 1.553398 | .7239347 | 1.543689 | .6970062 |
|  | Iss75 | 4.077670 | .8247319 | 4.106796 | .8033340 |
|  | Iss76 | 1.543689 | .5903920 | 1.533981 | .5742093 |
|  | Iss77 | 1.543689 | .6827956 | 1.524272 | .6393112 |
|  | Iss78 | 1.504854 | .6697056 | 1.485437 | .6240927 |
|  | Iss79 | 1.533981 | .6073978 | 1.533981 | .5910365 |
|  | Iss80 | 1.514563 | .6840490 | 1.495146 | .6397577 |
|  | Iss81 | 1.669903 | .6475951 | 1.660194 | .6031518 |
|  | Iss82 | 1.504854 | .6549029 | 1.485437 | .6240927 |
|  | Iss83 | 1.563107 | .6366256 | 1.553398 | .5895854 |
|  | Iss84 | 4.058252 | .8022669 | 4.058252 | .8022669 |
|  | Iss85 | 1.689320 | .7005478 | 1.650485 | .6056715 |
|  | Iss86 | 1.524272 | .7118694 | 1.495146 | .6242452 |
|  | Iss87 | 1.553398 | .6675703 | 1.533981 | .5742093 |
|  | Iss88 | 1.495146 | .6397577 | 1.485437 | .6240927 |
|  | Iss89 | 4.135922 | .7675853 | 4.145631 | .7593569 |
|  | Iss90 | 1.514563 | .6081808 | 1.514563 | .5918412 |
|  | Iss91 | 1.660194 | .6500890 | 1.650485 | .6056715 |
|  | Iss92 | 1.572816 | .7222234 | 1.524272 | .5915195 |
|  | Iss93 | 1.553398 | .7505313 | 1.504854 | .6397577 |
|  | Iss94 | 1.563107 | .6518436 | 1.533981 | .5910365 |
|  | Iss95 | 3.961165 | .8736034 | 4.000000 | .8284169 |
|  | Iss96 | 3.902913 | .8461512 | 3.922330 | .8247319 |
|  | Iss97 | 1.650485 | .6524274 | 1.640777 | .6080243 |
|  | Iss98 | 1.504854 | .6697056 | 1.485437 | .6240927 |
|  | Iss99 | 3.970874 | .8683578 | 4.009709 | .8224205 |
|  | Iss100 | 1.495146 | .6242452 | 1.504854 | .6242452 |
|  | Iss101 | 1.601942 | .7321712 | 1.553398 | .6059858 |
|  | Iss102 | 1.582524 | .7346371 | 1.533981 | .6540303 |
|  | Iss103 | 1.582524 | .6934465 | 1.563107 | .6050426 |
|  | Iss104 | 1.679612 | .6890401 | 1.650485 | .6056715 |
|  | Iss105 | 4.000000 | .8966167 | 4.029126 | .8795756 |
|  | Iss106 | 1.582524 | .7074432 | 1.543689 | .6067706 |
|  | Iss107 | 3.852941 | .8832552 | 3.854369 | .8790343 |
|  | Iss108 | 1.873786 | .8004853 | 1.873786 | .7498969 |
|  | Iss109 | 1.582524 | .6934465 | 1.563107 | .6050426 |
|  | Iss110 | 3.941748 | .8725132 | 3.970874 | .8569933 |
|  | Iss111 | 1.504854 | .6697056 | 1.504854 | .6397577 |
|  | Iss112 | 1.533981 | .6388644 | 1.533981 | .6233297 |
|  | Iss113 | 3.970874 | .8683578 | 3.990291 | .8687962 |
|  | Iss114 | 1.495146 | .6549029 | 1.504854 | .6549029 |
|  | Iss115 | 3.912621 | .8977837 | 3.922330 | .8821689 |
|  | Iss116 | 1.592233 | .6778989 | 1.582524 | .6343790 |
|  | Iss117 | 3.932039 | .8315133 | 3.961165 | .7786657 |
|  | Iss118 | 1.524272 | .7255108 | 1.504854 | .6397577 |
|  | Iss119 | 3.796117 | .8895827 | 3.825243 | .8451382 |
|  | Iss120 | 4.000000 | .8966167 | 4.009709 | .8910794 |
|  | Iss121 | 1.533981 | .6073978 | 1.543689 | .6067706 |
|  | Iss122 | 1.475728 | .6393112 | 1.475728 | .6237876 |
|  | Iss123 | 1.689320 | .7279992 | 1.640777 | .6080243 |
|  | Iss124 | 4.019417 | .8399414 | 4.038835 | .7911561 |
|  | Iss125 | 1.504854 | .6697056 | 1.495146 | .6242452 |
|  | Iss126 | 3.951456 | .8674805 | 3.970874 | .8219574 |
|  | Iss127 | 1.563107 | .6812604 | 1.524272 | .5915195 |
|  | Iss128 | 4.009709 | .8224205 | 4.019417 | .8162634 |
|  | Iss129 | 4.019417 | .8742569 | 4.029126 | .8683578 |
|  | Iss130 | 1.553398 | .6375221 | 1.543689 | .5903920 |
|  | Iss131 | 1.553398 | .7373529 | 1.533981 | .6540303 |
|  | Iss132 | 3.961165 | .8736034 | 3.990291 | .8224205 |
|  | Iss133 | 4.067961 | .7953558 | 4.077670 | .7882634 |
|  | Iss134 | 1.582524 | .7736376 | 1.533981 | .6540303 |
|  | Iss135 | 1.563107 | .6812604 | 1.524272 | .5915195 |
|  | Iss136 | 1.519608 | .7275402 | 1.495146 | .6397577 |
|  | Iss137 | 1.514563 | .6840490 | 1.504854 | .6397577 |
|  | Iss138 | 1.572816 | .6802817 | 1.563107 | .6050426 |
|  | Iss139 | 1.699029 | .7253796 | 1.640777 | .6080243 |
|  | Iss140 | 1.592233 | .7062313 | 1.553398 | .5895854 |
|  | Iss141 | 1.524272 | .6837707 | 1.485437 | .6240927 |
|  | Iss142 | 4.009709 | .7982228 | 4.029126 | .7977457 |
|  | Iss143 | 1.708738 | .7089217 | 1.679612 | .6137891 |
|  | Iss144 | 1.572816 | .6355782 | 1.563107 | .6050426 |
|  | Iss145 | 1.524272 | .7255108 | 1.504854 | .6397577 |
|  | Iss146 | 4.067961 | .7829323 | 4.077670 | .7757264 |
|  | Iss147 | 1.582524 | .6934465 | 1.572816 | .6802817 |
|  | Iss148 | 2.029126 | .8569933 | 2.019417 | .8281870 |
|  | Iss149 | 1.737864 | .7404446 | 1.728155 | .6743795 |
|  | Iss150 | 3.980583 | .8629700 | 3.990291 | .8574374 |
| Average | |  | .72896708 |  | .68786044 |
