# Supplementary material for: Requirements engineering issues causing software development outsourcing failure
Source: PLoS One. 2020 Apr 9;15(4):e0229785. doi: 10.1371/journal.pone.0229785 (PMC7144980; doi:10.1371/journal.pone.0229785)
Supplement: Supplementary file 5 [file pone.0229785.s001.odt]

## S1 Literature assessment. Literature assessment details

## 1. Systematic literature review

Systematic Literature Review (SLR) has been conducted to identify the issues of Requirements Engineering (RE) process for Software Development Outsourcing (SDO) and to explore the relevant RE practices that can be  used to address those issues. This SLR has been carried out by using guidelines as given in the study ([Keele, 2007](#_ENREF_121); [B. Kitchenham, 2004](#_ENREF_130)).

1.1. Research questions

The aim of this SLR is to explore the existing literature to identify the issues of RE process for SDO and related RE practices to address those issues. Thus, primary research question for this SLR is “Which are literature-based issues of RE process in case of SDO and which are literature-based relevant RE practices to address those issues?” This primary research question has been decomposed into two research questions:

RQ1: Which are literature-based issues of RE process for SDO?

RQ2: Which are the relevant RE practices, reported in the literature, to address literature-based issues of RE process for SDO?

1.2. Data sources

Five Electronic databases (ED) have been employed as data sources to find potentially relevant studies. The selected electronic databases have been listed in Table 1. 

            Table 1: Electronic databases searched for systematic literature review

| Identifier | Database | URL |
| --- | --- | --- |
| ED1 | IEEE Xplore | [http://ieeexplore.ieee.org/](http://ieeexplore.ieee.org/) |
| ED2 | ACM | [http://dl.acm.org/](http://dl.acm.org/) |
| ED3 | Science Direct | [http://sciencedirect.com/](http://sciencedirect.com/) |
| ED4 | Springer Link | [http://link.springer.com/](http://link.springer.com/) |
| ED5 | Web of Science | [http://webofknowledge.com/](http://webofknowledge.com/) |

1.3. Search string

The following search terms have been used to define search string for searching the electronic databases. These search terms have been derived keeping in view the research questions and relevant literature.

- Requirements

- Issues OR problems OR risks OR errors OR challenges OR barriers

- Practice OR lesson OR solution OR strategy OR strategies

- Outsource OR outsourced OR outsourcing

- Domestic OR onshore OR onshoring OR “on shore” OR nearshore OR nearshoring OR nearshored OR “multi-site” OR offshore OR offshoring OR offshored OR “off shore” OR global OR distributed

- “Software development” 

The search string has been derived by combining these search terms, using conjunction (AND) and disjunction (OR) operators, and wildcard *. The resulting search string is:

((Requirements) 

AND

 (Issues OR problems OR risks OR errors OR challenges OR barriers OR 

practice OR lesson OR solution OR strateg*)  

AND 

(outsourc* OR domestic OR onshor* OR "on shore" OR nearshor* OR "multi-site" OR offshor* OR "off shore" OR global OR distributed) 

 AND 

("Software development"))

This basic search string has been applied in different forms to search the selected electronic databases. For example, to find the relevant studies from IEEE Xplore, the basic search string has been decomposed in to five sub-strings. These sub-strings have been applied one by one through the Command Search option. The sub-strings have been shown in Fig 1.

i)   ((Requirements)

AND

(Issues OR problems OR risks OR errors OR challenges OR barriers OR

practice OR lesson OR solution OR strateg*)

AND

(outsourc* )

AND

("Software development"))

ii)  ((Requirements)

AND

(Issues OR problems OR risks OR errors OR challenges OR barriers OR

practice OR lesson OR solution OR strateg*)

AND

(Domestic OR onshor* OR “on shore”)

AND

("Software development"))

iii)  ((Requirements)

AND

(Issues OR problems OR risks OR errors OR challenges OR barriers OR

practice OR lesson OR solution OR strateg*)

AND

(nearshor* OR “multi-site”)

AND

("Software development"))

iv) ((Requirements)

AND

(Issues OR problems OR risks OR errors OR challenges OR barriers OR

practice OR lesson OR solution OR strateg*)

AND

(Offshor* OR “Off Shore”)

AND

("Software development"))

v) ((Requirements)

AND

(Issues OR problems OR risks OR errors OR challenges OR barriers OR

practice OR lesson OR solution OR strateg*)

AND

(Global OR distributed)

AND

("Software development"))

 

Fig 1: Sub-strings for searching from IEEEXplore

 1.4.  Selection of studies

The studies’ selection procedure for SLR has been presented in Fig 2. This procedure encompasses four phases which are: i) Identification of studies, ii) Screening of studies on the basis of title and abstract,  iii) Screening of studies on the basis of full-text, and iv) Final selection. To avoid biasedness, two fellow researchers have been involved during all the phases and each phase has been completed after developing consensus.

Fig 2: Studies selection procedure

1.5. Inclusion and exclusion criteria

The inclusion and exclusion criteria have been applied during the studies’ selection procedure to choose the potentially relevant studies that are suitable to answer the research questions. Time period for automated searching from selected electronic databases is from January 2000 to June 2014. The searching has been performed in July 2014. The inclusion and exclusion criteria employed for this SLR are:

    - Inclusion criteria

The Inclusion Criteria (IC) for this SLR are: 

IC1: Studies that are related to the issues of RE process for SDO.

IC2: Studies that are related to the practices that can be used to address the issues of RE process for SDO.

    - Exclusion criteria

The Exclusion Criteria (EC) for this SLR are: 

EC1: Studies that are duplicated in more than one electronic database or duplicated in automated and manual search.

EC2: Studies other than English language.

EC3: Studies that are not related to the issues of RE process for SDO or are not related to the practices that can be used to address the issues of RE process for SDO.

EC4: Studies with ambiguous findings.

1.6.   Quality assessment criteria

The Quality Assessment (QA) criteria for this SLR are:

QA1: Are the inclusion and exclusion criteria appropriate?

QA2: Has impartiality been ensured during the selection of studies?

QA3: Are the studies context specific?

QA4: Is evidence in support of findings?

The inclusion and exclusion criteria have been defined and reviewed carefully keeping in view the research questions. To guarantee neutrality during studies selection procedure, two fellow researchers have been involved during all the phases of this procedure. Furthermore, only those studies have been selected for data extraction that are relevant to the context. For example, studies regarding insourced offshoring or insourced near shoring have been excluded. Lastly, only those studies have been considered for final selection in which evidences support the results and there are no contradictions.

Keeping in view inclusion & exclusion criteria, and quality assessment criteria, each study has been reviewed by three reviewers. After passing through an arduous review process and screening, finally 117 studies have been selected for data extraction. These 117 studies include 77 studies from automated searching and 40 studies from manual searching. Database-wise details of 77 studies, selected through automated searching, have been provided in Table 2.

Table 2:No. of retrieved studies and finally selected studied from the different electronic databases

| Identifier | Database | No. of retrieved studies | No. of finally selected studies | Percentages to total no. of finally selected studies |
| --- | --- | --- | --- | --- |
| ED1 | IEEE Xplore | 431 | 39 | 50.65≈51% |
| ED2 | ACM | 310 | 10 | 12.99≈13% |
| ED3 | Science Direct | 679 | 08 | 10.39≈10% |
| ED4 | Springer Link | 662 | 12 | 15.58≈16% |
| ED5 | Web of Science | 253 | 08 | 10.39≈10% |
| Total | 2335 | 77 | 100% |

Fig 3 represents the percentages of the finally selected studies from different databases.  

Fig 3: Database-wise percentages of finally selected studies

The 40 studies have been selected through the manual search that is based on searching the issues of RE process for SDO and practices to address those issues, publications of specific authors and references of the studies retrieved through automated searching.

These 117(77+40) studies have been investigated thoroughly to find the issues of RE process for SDO and to explore the relevant RE practices to address those issues. The issues are related to different categories like communication, knowledge management, culture etc. A questionnaire survey has been conducted to find the categories of the issues according to the perception of SDO practitioners.

References

Keele, S. (2007). Guidelines for performing systematic literature reviews in software engineering Technical report, Ver. 2.3 EBSE Technical Report. EBSE.

Kitchenham, B. (2004). Procedures for performing systematic reviews. Keele, UK, Keele University, 33(2004), 1-26.
