# Supplementary material for: Requirements engineering issues causing software development outsourcing failure
Source: PLoS One. 2020 Apr 9;15(4):e0229785. doi: 10.1371/journal.pone.0229785 (PMC7144980; doi:10.1371/journal.pone.0229785)
Supplement: Supplementary file 6 [file pone.0229785.s002.odt]

S2 Survey participants: Survey participants details and recruitment

- Participants details and recruitment method

Three questionnaire surveys have been conducted to complete the study. The guidelines given in the study ([B. A. Kitchenham & Pfleeger, 2008](#_ENREF_132)) have been followed to design and conduct the surveys. Before starting the surveys, we searched for such:

 i). Software development companies which dealt with outsourced software development,

 ii). Organizations/companies which outsourced the software development instead of developing inhouse. 

Keeping in view target companies/organizations, we identified 110 companies and organizations by making telephone calls, sending emails and searching websites. The identified organizations and companies were contacted at various levels and were requested to take part in the study. The representatives were informed about the study objectives and criteria for the participants. Finally, 63 companies were selected from Malaysia and Pakistan based on the responses received and business nature. The research was conducted in the Malaysia, and participants belonging to selected companies and organizations were engaged for surveys from August 2014 to December 2014 based on the inclusion and exclusion criteria.

1.1.Inclusion criteria 

The Inclusion Criteria (IC) for participants are:

IC1: Participant’s company or organization profile

The main business of the participant’s company must be dealing with software development outsourcing, or the participant must be from an organization or company which outsource/outsourced the software development instead of developing inhouse.

IC2: Participant’s job title

The participant must be a project manager, software engineer, team lead, quality assurance manager, programmer, designer, requirements engineer, analyst or operational manager, senior manager etc.

IC3: Participant’s outsourcing related job experience

The participant must have at least 5 years’ experience of outsourcing related professional job.

1.2. Exclusion criteria 

The Exclusion Criteria (EC) for participants are:

EC1: Irrelevant profile of the participant’s company or organization

The main business of the participant’s company is not dealing with the software development outsourcing, or the participant does not belong to an organization or company which outsource/outsourced the software development instead of developing inhouse.

EC2: Unsuitability of the participant’s job title

The participant’ job is clerical or administrative etc. 

EC3: Participant’s lack of outsourcing related job experience

The participants’ experience of outsourcing related professional job is less than 5 years.

EC4: [Incredibility](https://www.merriam-webster.com/dictionary/incredibility) of the participant’ s filled questionnaire 

All the required questions have not been answered, or answers of the two or more than two questions are conflicting.   

Demographic details of the participants have been provided in the Table 1.

Table 1. Demographic details of participants

| Sr # | Factor | Details |
| --- | --- | --- |
| 1 | Number of Participants | Survey # Number of participants Percentage To whom questionnaire were distributed From whom questionnaire received back Whose questionnaire selected for data analysis 1st         200         115 105 52.50% 2nd 200 110 106 53.00% 3rd(Delphi round 2) 118 110 110 93.22% 3rd(Delphi round 3) 110 106 103 93.64% |
| 2 | Respondents’  Job Titles | Project manager, software engineer, team lead, quality assurance manager, programmer, designer, requirements engineer, analyst, operational manager, senior manager |
| 3 | Respondents’ Outsourcing relevant experience | Minimum = 5 years  Maximum= 15 years |
| 4 | Number of respondents’ companies or organizations w. r. t. relevant profile | Companies or organizations dealing with software development outsourcing= 63 |
| 5 | Number of respondents’ companies or organizations w. r. t. number of employees | According to Malaysian SMEs Standard at that time: Companies or organizations with employees from 5-29     =   10 (Small) Companies or organizations with employees from 30-75   =   30 (Medium) Companies or organizations with more than 75 employees=  23  According to Pakistani SMEs Standard at that time: Companies or organizations with employees from 10-35     = 12 (Small) Companies or organizations with employees from 36-99     = 31 (Medium) Companies or organizations with more than 99 employees=  20 |
| 6 | Number of respondents’ companies or organizations w. r. t. business scope | National =       03 Regional =       15 International= 45 |
| 7 | Number of respondents’ companies or organizations w. r. t. certification | Certified (CMMI or ISO) = 55 Non-certified =                     08 |
| 8 | Types of the projects handled by companies or organizations | Embedded systems, Telecommunication systems, Business systems, E-Commerce systems, Multimedia applications, Web-based systems, Safety critical systems, Accounting and Finance Systems, Billing services Systems |

1.3 Why convenience sampling was conducive and 

why sample can be considered representative of a larger population

To attain the objectives of the study, three questionnaire surveys have been conducting whereas two of these surveys involve very lengthy questionnaires. Keeping in view nature of the study, we wanted that same participants or at least participants from the same companies or organizations should participate in the surveys to complete the study. Time constraints were also there. In these circumstances, we contacted with software development outsourcing practitioners or their representatives from various countries of the world. But the results were extremely disappointing as practitioners were busy or were not available at that particular time. Therefore, we had to adopt convenience sampling method. Through the convenience sampling those practitioners, from Malaysia and Pakistan, were included in the study who were willing to participate in the study upon our personal request or because of any academic or industrial reference. At the same time, for sake of quality, we ensured that:

i). All the participants belong to the companies or organization which deal with software development outsourcing.

ii). All the participants have at least 5 years’ experience of software development outsourcing related professional job.

iii). Participants belong to various professional categories like project manager, quality assurance manager, software engineer, team lead, requirements engineer, analyst, programmer etc.

iv). Participants have vast experience of dealing with a wide range of projects like Telecommunication systems, Web-based systems, Safety critical systems, Accounting and Finance Systems, Billing Services Systems etc.

v). Most of the respondents ‘companies or organizations are certified.

vi). Respondents’ companies or organizations vary in size from small to medium and large.

vii). The number of respondents in case of each survey is reasonable (more than 100).

Moreover, this study is part of PhD work. A model has been proposed based on the findings of this study. The model has been validated by the experts, with vast industrial and academic experience, from various countries. Thus, we can say that experts agreed with the findings or contributions of this study.

Based on all these facts, we can say that our sample(s) can be safely considered as representative of large population.

References

Kitchenham, B. A., & Pfleeger, S. L. (2008). Personal opinion surveys Guide to Advanced Empirical Software Engineering (pp. 63-92): Springer.
