# Supplementary material for: Requirements engineering issues causing software development outsourcing failure
Source: PLoS One. 2020 Apr 9;15(4):e0229785. doi: 10.1371/journal.pone.0229785 (PMC7144980; doi:10.1371/journal.pone.0229785)
Supplement: Attachment — Submitted filename: Response to Reviewers.odt [file pone.0229785.s005.odt]

Source of Comments
Comment #
Response to Comment


REVIEWER 1


Overall Comment: The paper seems technically sound and very intelligently written and hence is a good addition in the software engg domain. Following minor improvements are suggested to further improve the paper.

We appreciate the comment.

1: The title of the paper is too long and sounds ambiguous. The authors may consider to re-structure the title.
 The title has been changed. 

2: Throughout the text the author has not taken care of the capital and small letters.
The paper has been reviewed thoroughly and reviewer’s concern has been addressed. The changes have been highlighted.

3: Page 3, Line 51, "The RE related errors occur commonly during 62 SDLC [25]. According to an industrial survey, such errors are 48%" the statement needs to be justified. The context is not clear.
More information have been added to make the context clear

4: Page 15, Table 1. the table should also mention the % of finally accepted papers as compared to initially identified papers.
 A new column has been introduced in Table 1 to mention required percentage

5: Page 12, Table 2, the identified issues and shortfalls need to be justified.

Done as recommended.


6: Before conclusion section, the author should have a detailed discussion section.
Discussion section has been added.

7: Limitation and future work section needs to be added.
Limitations and future work have been added.


REVIEWER 2


Overall Comment: Author has performed an extensive and detailed study. He resulted with great outcomes.
Authors have done excellent job for Software development outsourcing failure: Identification, categorization and ranking of
the customarily occurring requirements engineering issues.
A detail Delphi process is adopted on extensive data set and a good quality of evidence were collected from literature too to support their claim.  


We appreciate the comment.


Journal Requirements
1: Please ensure that your manuscript meets PLOS ONE's style requirements, including those for file naming.
 
 
 Done

2: We suggest you thoroughly copyedit your manuscript for language usage, spelling, and grammar
 
Done, changes highlighted

2a: Provide the name of the colleague or the details of the professional service that edited your manuscript
 
Dr. Muzafar Khan

2b: Provide a copy of your manuscript showing your changes by either highlighting them or using track changes (uploaded as a *supporting information* file)

Provided as supporting information file 
“S5 Revised manuscript with track changes” 

2c: Provide a clean copy of the edited manuscript (uploaded as the new *manuscript* file)

Clean copy provided as file   “Manuscript”

3: Thank you for including your ethics statement
We appreciate the comment.

3a: Please amend your current ethics statement to include the full name of the ethics committee/institutional review board(s) that approved your specific study.
 
 

 Done

3b: Once you have amended this/these statement(s) in the Methods section of the manuscript, please add the same text to the “Ethics Statement” field of the submission form (via “Edit Submission”).

 
  
 Done
